# Unlocking Insights in IoT-Based Patient Monitoring: Methods for Encompassing Large-Data Challenges

**DOI:** 10.3390/s23156760

**Published:** 2023-07-28

**Authors:** Muhammad Waleed, Tariq Kamal, Tai-Won Um, Abdul Hafeez, Bilal Habib, Knud Erik Skouby

**Affiliations:** 1 Department of Electronic Systems, Aalborg University Copenhagen, 2450 København, Denmark; skouby@es.aau.dk; 2 Electrical and Computer Engineering, Habib University, Karachi 75290, Pakistan; 3 Graduate School of Data Science, Chonnam National University, Gwangju 61186, Republic of Korea; 4 Computer Science and Applications, Virginia Tech, Blacksburg, VA 24061, USA; 5 Department of Computer Systems Engineering, University of Engineering and Technology (UET), Peshawar 25120, Pakistan

**Keywords:** remote patient monitoring, internet of things, edge processing, cloud computing

## Abstract

The remote monitoring of patients using the internet of things (IoT) is essential for ensuring continuous observation, improving healthcare, and decreasing the associated costs (i.e., reducing hospital admissions and emergency visits). There has been much emphasis on developing methods and approaches for remote patient monitoring using IoT. Most existing frameworks cover parts or sub-parts of the overall system but fail to provide a detailed and well-integrated model that covers different layers. The leverage of remote monitoring tools and their coupling with health services requires an architecture that handles data flow and enables significant interventions. This paper proposes a cloud-based patient monitoring model that enables IoT-generated data collection, storage, processing, and visualization. The system has three main parts: sensing (IoT-enabled data collection), network (processing functions and storage), and application (interface for health workers and caretakers). In order to handle the large IoT data, the sensing module employs filtering and variable sampling. This pre-processing helps reduce the data received from IoT devices and enables the observation of four times more patients compared to not using edge processing. We also discuss the flow of data and processing, thus enabling the deployment of data visualization services and intelligent applications.

## 1. Introduction

A general healthcare system involves preventing, controlling, managing, and treating sickness, disease, injury, or disability and also requires addressing the care and aftercare of the person with these needs, which may or may not be carried out by a healthcare professional [1]. The need for patient care (requiring constant monitoring and checkup) has increased many-fold due to several reasons, such as (i) the intake of unhealthy and fatty foods, smoking, stress, and inactivity; (ii) increase in life expectancy as a larger population is in the old age group [2]; (iii) increase in vascular disease and diabetes; and (iv) large-scale pandemics such as COVID-19, influenza, and Hepatitis C [3], among other factors. Further, the shift in culture and policy adjustments have contributed to a more patient-centered and costly healthcare system over the last century. Hospital care incurs enormous expenses and is often overburdened. According to George Washington University’s report, the use of hospital services is expected to grow significantly, causing a rise in healthcare industry costs from 0.9 percent of GDP in 2019 to 2.4 percent by 2025 [4]. Cutting-edge technology needs, therefore, to play an increasingly significant role in our healthcare system. Many insurance companies are investing in the adoption of proactive approaches to detect health issues early and avoid hospitalizations [5,6]. One way to reduce hospitalizations and improve healthcare is to move from constant monitoring in health facilities to remote patient monitoring. The shift is significant, as technological advancements in healthcare electronic technologies, such as sensors, wearable devices, and GPS tracking, are tested.

The internet of things (IoT) is emerging as a relevant choice for remote monitoring and sensing. It refers to the interconnection of sensing devices that facilitates a coordinated activity, which enables the monitoring, control, and calibration of a task or an activity [7,8,9]. Modern IoT systems find massive use in applications such as urban monitoring [10], emergency services, traffic management, waste management, building monitoring, transportation, industries, and healthcare [11,12]. IoT is also finding ample use in the monitoring and treatment of chronic diseases using custom fitness programs [13,14], care of elderly persons [15], and several medical sensing devices, recorders, and image diagnostic equipment play a central part in IoT to help make intelligent and informed decisions [16]. The IoT-supported patient monitoring and remote telemetry aim to improve the healthcare system and reduce its associated costs [17]. Current systems generally discuss individual aspects of the monitoring frameworks; however, to fully utilize the promise of IoT and associated technologies (cloud, machine learning, communication, etc.), an end-to-end solution is vital. This will require a scalable model at the sensing, network, and cloud levels and their well-defined integration.

In this paper, we propose a scalable cloud-based model for the remote monitoring of patients. The model consists of three well-defined modules: sensing, network, and application. The sensing module uses IoT devices to collect the patient’s data. The data are continuously transferred to the cloud using API gateways. The network module uses cloud services and consists of several subsystems. The sensing module’s data are provided to the network module, which stores and feeds them to services such as AI, machine learning, visualizations, and decision making. This module has several data processing and notification services. The application module provides an interface for the end users using desktop, web, and mobile applications and supports the end user in visualizing the data and performing remote interactions.

The proposed model is designed to handle large-scale data collected from IoT devices mounted on a limited number of people (as lab experiments) by applying filtering, variable sampling and multiplexing at the edge level (IoT devices and intermediate hub). The results show that the model can support four times more patients by reducing the data burden through edge processing techniques. Besides supporting the increased number of patients, the gains are realized at the network layer by reducing pressure on the cloud services and network bandwidth. In the network module, the data are stored in relational and non-relational storage to enable the application of services like machine learning and AI. The well-defined restful APIs enable the interaction between cloud services and user applications.

The rest of the paper is laid out as follows. Section 1.1 highlights the scientific contributions of this work. Section 2 describes the related work, followed by the proposed methodology in Section 3. Section 3.1, Section 3.2 and Section 3.3 introduce the sensing, network, and application modules of our model, respectively. Section 4 details the tools used for implementation, followed by Section 5, which presents the experiments and evaluation. Finally, Section 6 serves as a comprehensive discussion and conclusion of our proposed model, highlighting its key features, advantages, and potential applications. We also provide insights into future research directions and potential areas of improvement.

### 1.1. Contributions

The uniqueness of our model stems from addressing different aspects of remote patient monitoring that handles significant data burden at different levels, addresses the integration and archiving of data, enables smart diagnostic services, and integrates different agents.

The contributions of this work are listed as follows:

- An end-to-end system for remote patient monitoring that shows the collection, transmission, processing, archiving and application of data is developed.
- A modular and layered approach to patient monitoring is presented, which enables futuristic technology-driven (AI and ML) healthcare services.
- Edge processing is deployed to reduce the flow of ample data into the system.
- Well-defined APIs are incorporated, which enable the integration of IoT data with cloud services.
- Relational and non-relational data stores are included for the archiving of different data and information in the system.

## 2. Related Work

In this section, we survey a number of remote patient monitoring (RMS) systems and summarize their features and limitations. An overview is shown in Table 1.

The IoT has found widespread application in healthcare, particularly in health monitoring [18,19], and is mainly used for remote healthcare, telemetry, and control [20]. With the recent pandemic of COVID-19, the world is struggling to improve health services and incorporate cutting-edge technologies to fill the emerging gaps [21]. The 5G-based IoT systems effectively provide a way to monitor the patient without physical contact and enable health workers to evaluate the patient’s status [22]. The framework proposed by Saji et al. uses gateways to send data from biomedical sensors to the intermediate hubs for further processing [23]. The system is good at defining a gateway approach at the IoT layer, but fails to present all layers of the system. The system is not deployed in real clinical scenarios. Moreover, it does not provide the fundamental techniques for handling extensive data that the IoT generates or supports for heterogeneous devices. As IoT is widely experienced in health systems, smart navigation systems were developed for patients, allowing them to follow the ambulance in emergencies. This provides the location of patients to the hospital [24]. This system helps generate and enable early response. However, it fails to support larger applications and databases.

The IoT-supported system is helpful when patients are being treated at the hospital. Akira-Sebastian Poncette et al. proposed an IoT system for monitoring patients in the intensive care unit (ICU) that supports real-time alerts about the health of critical patients [25]. The system provides a good interactive environment, including hints on hardware and services. The system is still in the testing and validation phase and has not been deployed for clinical use. Further, work has yet to be conducted to upgrade it by adding new pressure and body weight calculation sensors. The model presented by Diwakar tracks the patient’s length of stay to manage the patient’s admission to the hospital efficiently [26]. The system is tested in the lab and can provide a health history and treatment information to assist doctors quickly.

The development of wearable devices enabled the IoT growth in the healthcare and biomedical fields. The biomedical sensors collect data and send them to the internet for further analysis and decision making [27]. The mounted devices have sensors and microcontrollers; sensors receive essential readings about the patient’s condition and send them to the nearby hub or smartphone using Wi-Fi or Bluetooth. There is no information provided regarding the clinical deployment of this system. Based on the type of sensors, the patient-monitoring wearable systems can be classified into four major categories: biomedical sensors, biopotential sensors, environmental sensors, and movement sensors [28]. The wearable IoT sensors (i.e., ECG) have found a significant impact in tackling cardiovascular diseases [29], and by using the wearable devices (e.g., mounting pulse sensors), the abnormality in the heart rate can be detected [30]. The working phenomenon is that the device alerts the caretakers to take action if an irregularity is detected. The pulse and ECG sensors can detect early cardiac arrest symptoms as Majumder et al. showed in their work [31]. The same was shown by Brezulianu et al. using IoT, signal processing and machine learning techniques [32,33]. These three systems are lab tested and have not been deployed for real clinical use.

IoT has found further application in measuring vital signs in patients, such as the respiratory rate, temperature and counting of breaths and their intensity [34]. A smart prototype developed in the lab for asthma patients was also shown using IoT sensors [35]. It uses watermarking and signal enhancement techniques for secure data transmission. The techniques reviewed by Lamonaca show blood pressure (BP) measurements using pulse, ECG, temperature, and respiratory sensors. It also showed the limitations of commercial BP measuring wearable IoT devices in measuring BP [36]. In a recent study, a novel three-factor authentication protocol for wireless body area networks (WBANs) was developed, integrating patient biometrics, smart card, and password, substantially enhancing security and performance [37]. Rigorous analyses employing real-or-random (ROR) and Burrows–Abadi–Needham (BAN) logic validated the protocol’s security, and it withstood the Dolev–Yao (DY) and Canetti–Krawczyk (CK) threat models. The protocol achieved a 43.98% reduction in computational overhead, an 18.18% increase in supported security characteristics, and a 19.05% reduction in space complexities. Another noteworthy study in wireless healthcare networks addressed security and privacy by developing an authentication scheme that ensures strong mutual authentication, backward secrecy, session key negotiation, and forward key secrecy in transmitting sensitive patient data over wireless channels [38]. The scheme proved resilient against various attacks and exhibited lower computation and communication complexities. However, both these studies and others fell short of providing a comprehensive, integrated model encompassing different layers of the system, which is essential for IoT-based remote patient monitoring.

**Table 1:** Comparative analysis of existing health monitoring systems with the proposed model.

Reference	Proposed Technique	Findings	Limitations
Serhani et al., 2017 [2]	Resource-Aware Mobile-Based Health Monitoring	Use of dynamic programming for finding the processing unit which reduces processing cost	No reduction in data generated from IoT, No mechanism for integration of AI/ML applications
Arora et al., 2019 [39]	Wearable Sensors Based Remote Patient Monitoring using IoT	Collection of patient data using IoT device and its upload to cloud	No reduction in IoT data, No cloud processing/archival, No support of future applications
Mohammed et al., 2014 [40], Raj 2020 [29], Wang et al., 2015 [33]	Monitoring of cardiovascular patients	Visualizes ECG and uploads IoT data to cloud	No reduction in collected data, can handle only one parameter (ECG)
Sangeethalakshmi et al., 2021 [13], Saji et al., 2021 [23], Diwakar et al., 2021 [26]	Real time health monitoring system using IoT	Records patients data and send it to cloud and physician app	No mechanism for handling of large IoT data, No direction about AI/ML services in cloud
Gomez et al., 2016 [14], Zhou et al., 2017 [15], Sharma et al., 2021 [27]	IoT-based fitness system	Employs IoT and cloud for data collection and processing	Focuses on one application, No filtering applied on IoT data
Mamatjan et al., 2016 [17]	Framework for RPM using a Wearable Device and Cloud	Integrates ML services in cloud with IoT data	No filtering is applied to reduce generated data
Javaid et al., 2021 [21], Paganelli et al., 2021 [22]	IoT enabled healthcare for COVID-19 Pandemic	Proposes a framework for collecting and processing data from COVID-19 patients	No implementation given, No data reduction techniques discussed
Poongodi et al., 2021 [24]	Smart healthcare in smart cities IoT applications	Interesting use of GPS to integrate ambulance service with hospital and patient	Very application specific, No support for cloud processing
Poncette et al., 2022 [25]	RPM System for Intensive Care Medicine	Usability study to evaluate UI experience of a remote patient-monitoring system	Do not develop or propose a RPM
Pasluosta et al., 2015 [28]	Overview of IoT for Management of Parkinson	Review the current sensors and technology	Do not develop the RPM system
Jayanth et al., 2017 [30]	Wearable device to measure heart activity using IoT	Uses pulse and ECG sensors to monitor heart condition and generate alerts	Localized, No handling of large data, No clear role of cloud servers
Milici et al., 2017 [34]	IoT-based system for people with breathing difficulties	Monitors breathing and generate alerts, store data on cloud	Localized to one application, No data filtering or multiplexing
Noda 2019 [41]	Wearable NFC and Sensor Tag for RMS	Experiments with first implementation of body flexible sensors	Uses data in a localized context, RMS not proposed
Yang 2014 [42]	A spatiotemporal compression for big data processing on Cloud	Use of compression and spatial information for the handling of large data	Proposed techniques focus on graphs, No study performed on streaming IoT data
Malensek et al., 2017 [43]	Fog and Cloud Domains to Support Query Evaluations in Sensing Environments	Uses decentralized edge processing to reduce data pressure in large systems	The system is not tested in a high volume and multiple nodes environment
Our proposed model	Cloud-based patient monitoring model to handle large data by deploying different edge computing techniques	Edge processing techniques for reducing data burden, significantly reduce data received from IoT devices, support four times more patients, enabling data visualization services	Deployment in real clinical scenarios, increase number of sensors and integrate AI and ML for more intelligent operations Note: These limitations are considered for future work

Frameworks that target the remote monitoring of patients have been proposed, including the evaluation of different components (i.e., data collection, AI, ML, alerts, processing, and applications); however, they fail to provide an overview of a coherent, integrated system. The model we offer is unique, as it explains the technical (and implementation) details of different aspects of a patient’s well-being in a remote or close setup. The model is described with characteristic features, like large data handling (collected from a limited number of people), cloud processing, adaptive sampling, and physician/guardian connection. We suggest that the given model enables a well-driven future healthcare system that could use/integrate state-of-the-art technology-driven (AI and ML) services.

## 3. System Description

**Figure 1:**
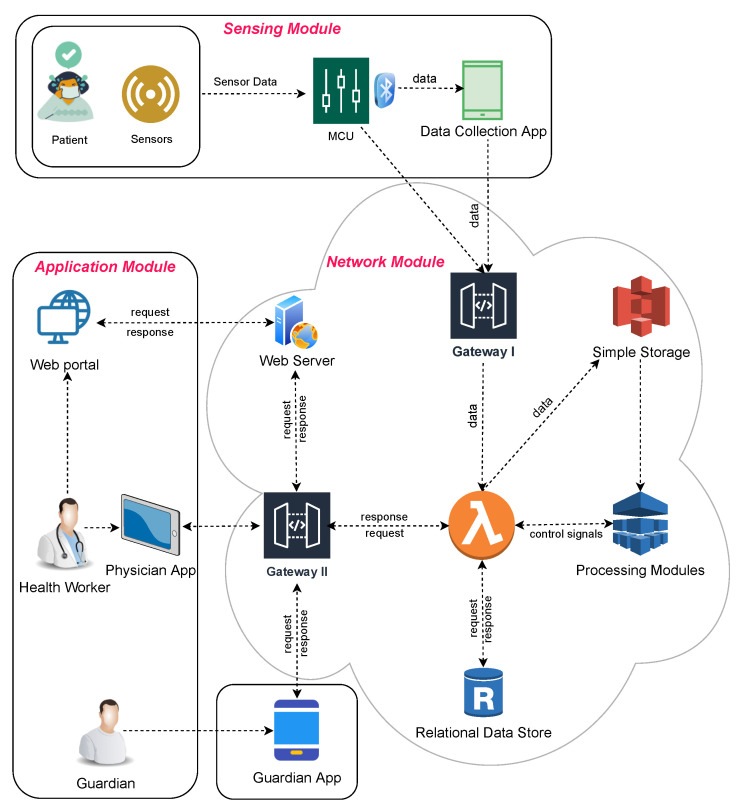
The model architecture diagram for remote patient monitoring. The sensing module collects data using IoT. The network module processes the data. The application module works as an interface for the end users. The modules are connected using well-defined APIs.

**Figure 2:**
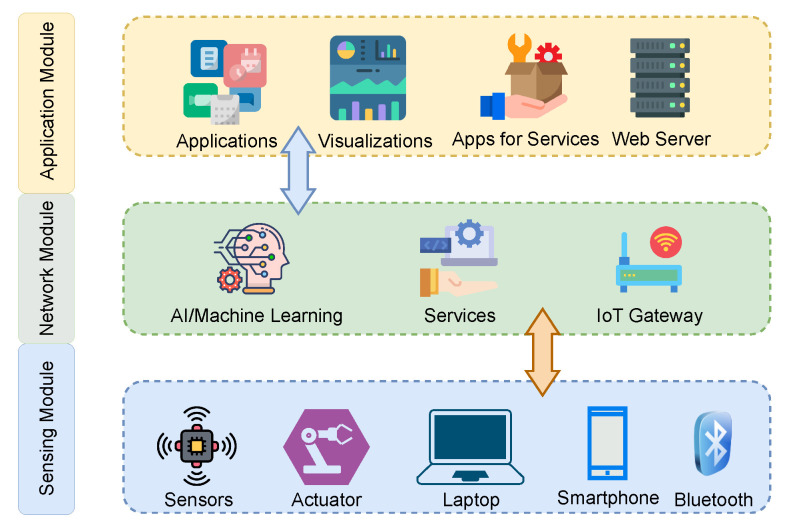
A block diagram showing the components in each module of the patient monitoring model. Each module is extendable, and new services/components can be added to it.

In this section, we outline the basic workflow of our proposed model as shown in Figure 1. The model comprises three main parts: sensing, network, and application. The sensing module, also known as the information-gathering part, collects data from IoT devices. This module records the patient’s vital signs and environment (temperature, pulse, movement, orientation, etc.) using sensor-mounted IoT devices and sends them to the cloud or the attached middleware (a smartphone or data multiplexing hub). The middleware connects to the controller on the IoT device via Bluetooth. The data from the sensing module are fed to the network module, which is implemented in the cloud and includes different services and API gateways. This module’s primary task is storing, managing, and processing incoming data. The application module, which contains the web and smartphone applications, acts as an interface for the guardians and physicians to interact with the model. The physician may define different interventions, notifications, and alerts through this module. Figure 2 shows the framework’s modular approach to services.

In the following three sections, we give details of the three modules of our model.

### 3.1. The Sensing Module

As shown in Figure 1, the sensing module uses IoT devices to record the patient’s data (temperature, pulse, movement, humidity, etc.) and sends them to the cloud via middleware. The data from sensors are gathered into the IoT device, and after applying some pre-implemented initial processing (filtering), they are sent to the smartphone app via Bluetooth. The smartphone app transfers the received data to the cloud through the API gateways (restful APIs). In some cases, like in the case of Sensor Tags or gadgets, where the device has a built-in WiFi module, the data may be directly sent to the cloud bypassing the middleware.

#### 3.1.1. Data Collection Using IoT Devices

The IoT is a network of small electronic devices with several sensors attached to a microcontroller. The sensors are placed on the patient’s body to measure the pulse, blood pressure, electric activity in the heart, muscle fatigue, brain signals, body temperature, skin hydration, breathing rate, blood oxygen, sound, and blood glucose [41]. The IoT is also helpful in finding the elevation, change in direction, physical movement, and GPS location. The device is equipped with Bluetooth, UART, or Wi-Fi to take the data out of the device into other computers, smartphones, or the cloud.

#### 3.1.2. Handling of Large IoT Data at Edge

Depending on the number of devices (and the number of attached sensors with each device), the generated data could be big, leading to pressure on the model due to several reasons:

1. A large amount of data creates processing pressure on middleware devices or intermediate hubs. The middleware often involves low-power devices (smartphones, etc.), and processing extensive data over a long period becomes more challenging.
2. The network or internet has bandwidth limitations, and injecting large data into it could result in data loss.
3. The extensive data could easily congest the cloud gateways, making them unavailable for further requests.
4. Processing a large volume of data needs extensive computing resources and smart load balancing, leading to increased handling, management and processing costs.

We apply filters at the edge (also called fog computing) to reduce the volume of data from IoT sensors. Table 2 shows the temperature readings and several other sensors (mounted on people in lab). Carefully looking at the data, we notice that most data points are redundant. In the table with 14 samples, the temperature value changes only six times in a range of 36.36–36.49, while the humidity changes only five times in a range of 20.9–21.5. This opens up two filtering opportunities: variation in sampling frequency and dropping of repeated readings.

The samples were collected at fixed intervals at about one millisecond apart. The same sampling frequency is often desired to ensure that the readings are synchronized in time and sent together to the middleware. However, using the same frequency for all sensors may result in redundant readings (some indicators, like temperature, do not change their values frequently). Therefore, the sampling frequency could be different for different sensors depending on the type of measurement and urgency. Our model uses a weighted system (in the range of 0–1), where the weights are assigned to sensors based on variations in their readings. The sampling frequency of a sensor is a factor of the sensor weight as shown in Equation (1):

(1) $$sensor_{samplingfrequency}=frequency_{maximum}*sensor_{weight}$$

where $frequency_{maximum}$ is the maximum frequency that can be achieved using a sensor. The maximum frequency is defined for each sensor. This means that a sensor with a higher weight is read more often than the sensor that carries a lower weight. The weight assigned is a combination of the sensor’s sensitivity and the patient’s condition as shown in Equation (2):

(2) $$sensor_{weight}=sensor_{sensitivity}*patient_{condition}$$

The sensor’s sensitivity and the patient’s condition are assigned numbers in the range of 0–1. $sensor_{sensitivity}$ is the sensitivity of the sensor. For example, the ECG readings are more sensitive, which change more frequently, showing lesser redundant values. In comparison, sensors for body temperature are less sensitive and may show redundant values if recorded at a higher frequency. $patient_{condition}$ shows the importance of a particular vital sign for a patient. For example, for a patient that is infected with COVID-19, the oxygen saturation level might carry more weight than the temperature.

The sensor sensitivity is a relative value, and its values can be set in two ways: static assignment and adaptable. In static assignment, the values are statically set once in the IoT device, given the standard variation values of a vital sign. For example, the temperature is less sensitive (in terms of variation from reading to reading) and could be set to a small value. The adaptable scheme could follow a more situation-aware mechanism and adjust the sampling frequency accordingly. For example, when a patient is asleep and static, the movement stops, and the gyro sensor show redundant or near-redundant values. In that case, the sensor sensitivity could be adjusted during operation.

The patient condition is also a relative value and a measurement of the importance of vital signs for a patient as perceived by the healthcare provider. If a physician or healthcare provider marks a sign as more vital (for example, the pulse readings as the condition of a heart patient worsens), its relative weight increases according to the increase in importance value by the healthcare providers. This quantitative value could also be driven by more informed techniques (algorithms, AI, and ML) based on the condition of the patient.

A second filter can be applied to gain more reduction in data volume. We notice that even when the sampling frequency is reduced, there may be room for duplicate or near-duplicate entries as is seen in Table 3. We apply a simple edge operation at the microcontroller level, where a data sample is dropped if the reading is the same (or falls within the threshold) as the previous reading from the sensor.

#### 3.1.3. The Smartphone App

As part of the sensing module, we developed an Android smartphone application that receives data from the IoT device via Bluetooth and applies some filtering (pre-processing) to it. The pre-processing checks the incoming data and drops the reading if they are not precarious. For example, obtaining a temperature reading in the range of 25–33 °C is considered normal. Alternatively, if deemed critical (i.e., patient pulse crosses 90), a data reading will generate alerts and notifications. In some cases, the data may not be immediately required until the entire sample is ready, such as the heartbeat signals recorded using a stethoscope; the stethoscope data help perform AI operations in the cloud. Further, the data are stored locally and sent over when the entire sample is recorded. The local cashing is helpful, as it allows for multiplexing (sending large packets, often as files), reducing the cloud gateways’ load. The smartphone may also facilitate specific sensors’ start/stop options to control the data flow.

At the sensing module, the gains at the microcontroller level (adaptive frequencies and duplicate removal) and the smartphone app level (local processing, multiplexing, and start/stop) help reduce the volume of large data. The gains are valuable in accommodating more devices, patients and cloud services.

### 3.2. The Network Module

The network module works at the cloud layer and collects data from the sensing module, performs its management, archiving, and further processing. The network module receives data through API gateways connected to the asynchronous event-driven servers. Depending on the data type, the receiving application may apply an initial check before sending them to the data store or other cloud services. The network module is further divided into four sub-parts based on functionality; details of each sub-part are given as follows.

#### 3.2.1. API Gateways for Sensing Module

The data from IoT devices or middleware (smartphone apps, etc.) can be received through API gateways in the cloud. The gateways are connected to the transport protocols. There are three options for receiving the incoming data. One is using the MQTT (message queuing telemetry transport) protocol; MQTT is a lightweight, publish–subscribe network protocol that helps carry messages between IoT devices and the cloud. The second option is to use streamers. The streamers are connected to producers and consumers and help carry large amounts of data. The producers push data to streamers asynchronously, and then the consumer receives data, typically multiple messages at a time (depending on the shard size). The third option is to use asynchronous event-driven services. The services are hosted on servers, which spin as a message (data) is received through the API gateway. We prefer event-driven services over MQTT and streamers, as it gives us more control over handling received data.

Further, the calling of micro-services (filtering, storage, AI, ML, visualization, etc.) is supported in the event handlers. The event handler also helps implement the authentication and verification mechanisms. After receiving, the data are checked for authenticity using the authentication services (JSON Web Tokens, etc.). All data exchanged through our APIs adhere to the JSON format. In terms of standards, we support HL7, a widely recognized standard in the healthcare industry. Our data-exchange practices align with HL7 compliance, ensuring compatibility and interoperability with other healthcare systems and applications. By adopting HL7 standards, we aim to promote seamless data integration and facilitate effective communication among healthcare stakeholders. We believe that verification mechanisms are essential in applications such as telemedicine, as they may involve sharing critical health information. If deemed necessary, a further layer of protection can be applied using encryption techniques only if it does not increase the computing time.

The event-driven services often perform a quick review of the data. If a flag is raised (occurrence of a severe condition requiring urgent medical care), an action is triggered, which involves sending notifications to healthcare professionals or controlling automatic treatment (like a supply of insulin if the glucose level rises above threshold).

#### 3.2.2. The Cloud Data Store

The cloud data store is divided into two parts: one for strong relational data and one for storing non-relational records. The relational data store is hosted on an SQL server that consists of databases, views and reports. The tables store the historical and current records of patients. Access to data on the relational instance is provided using separate gateways to ensure that the security is not compromised. The non-relational store archives the data in the form of files. It typically stores large, bulky data, such as audio clips and streaming data. Data for post-processing modules (AI, ML, etc.) are often fetched from this data store. The data store is extendable and could be easily integrated with hospital MIS (management information system).

#### 3.2.3. Data-Processing Applications

This module consists of several services that run in the cloud. The services run over the IoT data, and its purpose is to support physicians with aided diagnosis. Typically, the services incorporate machine learning and AI approaches. The services are triggered as new data are received about a patient. The range of services in this module can be large, and depending on the IoT data, more services can be added as needed. For example, the ECG data and heartbeat signals from a stethoscope can be used to develop a variety of ML and AI for aided diagnosis. Similarly, visualizations can be added to help healthcare providers/physicians’ study (amplify) selective parts of signals or imagery. Our proposed model is extendable, and different services can be added, such as AI-assisted heart diagnosis, etc. Similarly, the data on the cloud allow for the research and analysis of patient records. Although AI and ML modules were not implemented as part of this work, our model supports their addition. This extension is supported by adding more sensors (i.e., ECG for heart diagnosis) and services like machine learning classification models in the cloud. In AWS, the ML models are usually supported by services like Sagemaker.

#### 3.2.4. API Gateways for Application Module

To support the communication of the application module with the services on the cloud, we implement several API gateways. The gateways are attached to the stateless event-driven server to implement appropriate routing or actions. The communication from an application is authenticated using JSON Web Tokens (JWT). After authentication, the relevant service is called to respond to the request.

### 3.3. The Application Module

The application module provides the guardian and healthcare staff a medium to interact with the model. It consists of web dashboards and smartphone apps (web-based view, guardian app, physician app, etc.). The caretakers receive real-time information about the patient’s condition through these interfaces. Healthcare workers also use these interfaces to visualize the patient’s data and perform diagnoses assisted by AI and machine learning. These apps access data or services in the cloud through the API gateways of the application module. More details about the interfaces are given as follows.

#### 3.3.1. The Caretaker Smartphone App

This app provides an interface for the guardian or patient’s caretaker to interact with the model. The guardian receives real-time updates about the patient’s condition using this app. The app supports notifications to recieve alerts if the patient needs immediate attention.

#### 3.3.2. The Physician Smartphone App

This app is an interface for the healthcare provider/physician to interact with the model. The interface helps provide real-time alerts about the patient’s condition. The physicians use this to visualize the patient’s data. Moreover, they use it to calibrate the model, launch diagnosis applications in the cloud and increase or decrease the frequency of remote sensing. The physician also uses this app for remote telemetry.

#### 3.3.3. The Web Dashboard

The web dashboard is an authentication-controlled web interface. This interface can be used to perform visualizations and set up computer-aided diagnoses. The physicians can record their input on the patient’s condition through this interface. The interface can be opened for the guardians/caretakers to see the latest notes about the patient.

## 4. Implementation

This section shows an implementation of our proposed remote patient monitoring model. We will start with the implementation of the sensing module and then discuss other modules as well.

**Figure 3:**
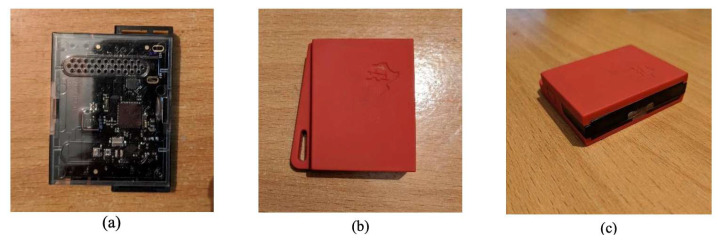
The TI CC2650 Sensor Tag IoT module. (**a**) Circuit clearly visible with cover removed, (**b**) view from the top with cover intact, (**c**) side view with on/off button [44].

The IoT device was implemented using the TI sensor Tag CC2650 (https://www.ti.com/tool/TIDC-CC2650STK-SENSORTAG (accessed on 15 December 2022)) as shown in Figure 3. The IoT Kit has ten sensors (temperature, light, elevation, direction, physical movement, humidity, atmospheric pressure, etc.). Each IoT tag is given a unique ID called the device ID. The IDs help in uniquely identifying the device and patient. Each patient is assigned exactly one device. The device ID is transmitted as part of the multiplexed data from the board. We apply the filtering on sensor data to reduce the pressure of data on the smartphone app, network, cloud gateways, and cloud services.

This section provides a detailed account of the implementation of our proposed remote patient monitoring model. We will begin by discussing the implementation of the sensing module and then proceed to cover other modules.

To implement the sensing module, we utilized the TI sensor tag CC2650, which served as our IoT device as shown in Figure 3. This IoT kit is equipped with ten sensors, including temperature, light, elevation, direction, physical movement, humidity, and atmospheric pressure, among others. Each IoT tag was assigned a unique device ID, enabling the precise identification of both the device and the corresponding patient. It is important to note that in our study, we tested with BLE-enabled IoT devices. This allowed us to perform multiplexing/edge processing at the intermediate device level. However, the system has well-defined APIs (post/get) that can handle data from any communication medium, like Bluetooth and Wi-Fi. The filtering and multiplexing techniques that are applied at the edge are important in reducing the volume of data generated from IoT devices. This ensured that only relevant and necessary data were transmitted and processed, optimizing the overall system performance and efficiency.

**Figure 4:**
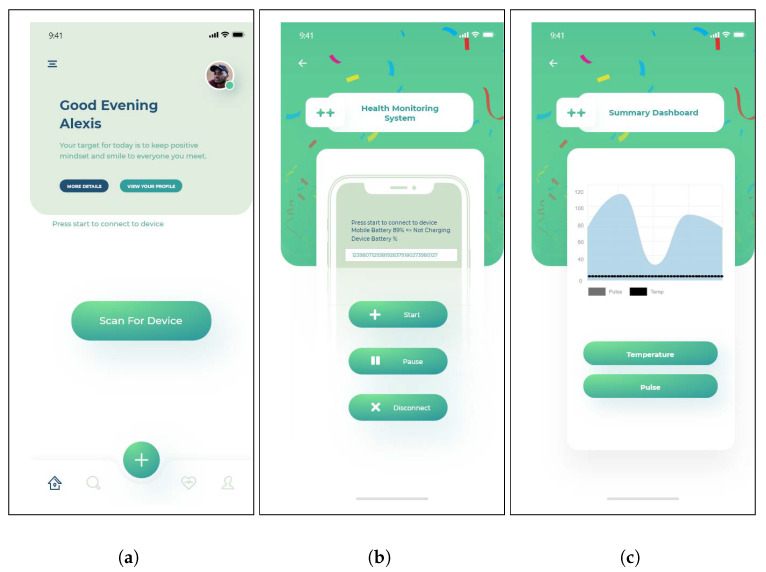
Overview of the cloud-based patient monitoring model architecture, illustrating the flow of data from the IoT sensors to the cloud for processing and analysis. (**a**) Scanning for available IoT devices using Bluetooth, (**b**) shows the data received from IoT device after being connected, (**c**) physician using the app for visualization.

The smartphone app was programmed in the React Native framework. The different interfaces of the app are shown in Figure 4. The android app receives data from the Sensor Tag via Bluetooth and applies a filter on it at the smartphone level. After filtering, the data are multiplexed into packets. The multiplexing helps reduce the number of outgoing data packets and the costs associated with the API calls. Further, the data packets are sent to the cloud using the cloud gateway. The Android app screenshot shows options for scanning, starting, and stopping the IoT device, where the incoming data can also be seen as shown in Figure 4.

The network module is provided in Amazon Web Services (AWS). The API gateways that are supported by Python functions handle the data coming into and out of the cloud. The Python functions run on the lambda server, an event-driven server that is stateless and enables asynchronous data handling. We implemented several Python functions for data checking, removing anomalies, and pushing the data to relational and non-relational data store. We also implemented a function for calling mock machine-learning models on Sagemaker (a machine-learning service in AWS).

For the relational data store, we launched a relational data service (RDS) instance in AWS and created a database (with relevant tables) using MySQL. The bulky sensor data are stored in the non-relational data store implemented in AWS S3 (simple store service), and the visualization is provided at the Android app level, programmed in the React Native framework.

The application module consists of a web dashboard, a physician app, and a guardian app. The web dashboard was programmed in HTML, Javascript, and CSS, while the Android apps were programmed in a React Native framework. The notification services were implemented using the AWS simple notification service (SNS).

## 5. Experiments and Evaluation

This section shows our model’s ability to remotely monitor patients using IoT, cloud and edge processing. It also evaluates its performance in handling large IoT data. We are interested in the following:

- The use of IoT devices in recording patient’s health and environment;
- The application of variable sampling and filtering in reducing the volume of data;
- The role of the data-collection app in receiving data from IoT, reducing the data volume using filtering and multiplexing, and data publishing to the cloud;
- The role of the cloud gateway in receiving the incoming data and triggering the handlers on an event-driven server;
- The role of non-relational and relational data stores in the archiving of data;
- The application of the guardian app in receiving notifications and the physician’s app in loading visualizations.

### 5.1. Experimental Setup

We perform our experiment with a different number of students (5–20) to validate the model’s functionality as shown in Table 4. Each student carries one IoT device, a Texas Instrument CC2650 IoT Sensor Tag. The IoT Kit carries ten sensors (light, barometer, magnetic sensor, humidity, pressure, 3-axis accelerometer, 3-axis gyroscope, magnetometer, object temperature, and atmospheric temperature). The data from the IoT device were recorded using variable frequencies (1000 samples per second and 500 samples per second). The sampling frequencies of 1000 and 500 do not follow some indicators’ conventions. For example, temperature and light do not change frequently and are typically recorded at a much smaller rate (one reading per minute) than this frequency. However, we performed this oversampling of data from different resources (e.g., sensors, and no access to hospital facility) to show the model’s applicability in processing extensive data (which crosses the bandwidth marks) and show the effects of edge processing. This was also performed to show that even if we only tested the model with a very limited number of patients, the volume of data is not a limiting factor (still our model has to be tested with a large number of patients). Several people used oversampling in several relevant applications to show the effect of significant data on the network and computing resources [42,43]. The ranges were chosen carefully only for testing the model, and the values may differ in different scenarios and geographical regions.

The work aimed to show the different aspects of a patient monitoring model and without pretending to emulate clinical analysis. Accordingly, the devices were mounted on people in our laboratory, not medical patients. The setup is a prototype not discussed/verified by medical experts; therefore, the patient parameters are taken here as an example, not necessarily being the most relevant/important. This is especially important in relation to the suggested parameters in Section 3.1 and Section 5.2. The clinical analysis is imperative, and we plan to perform that as part of future work, where we will also discuss with medical specialists different types of required variables when deploying the model in clinical scenarios.

### 5.2. Effect of Edge Computing at Device Level

To see the effect of edge computing, we mounted the IoT Sensor Tags on 10 people (posing as patients) as shown in Experiment 1 in Table 4. Each packet from the IoT device consists of 265 bytes (1-byte preamble, 4-byte access address, 257-byte PDU, and 3-byte CRC). Table 2 shows that the IoT module was able to record the status and environment using the 10 mounted sensors. When not applying any pre-processing and using a rate of 1000 samples per second, the device generates 21 Megabits of data per second. The 10 Mbps bandwidth could not support 10 patients; therefore, we experienced large delays and packet losses. With the given setup, a maximum of 5 patients could be supported on a 10 Mbps connection, although we encountered slight delays in data transmission.

**Figure 5:**
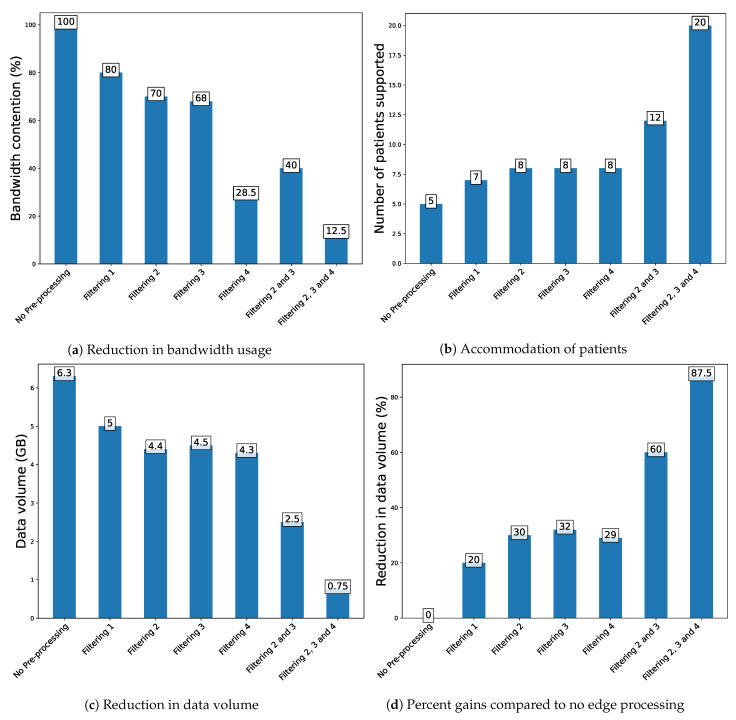
Comparing the gains from different filtering techniques when applied in standalone modes and in combination using a 10 Mbps network connection. (**a**) Data from 5 patients (when not applying any pre-processing), throttles the network resulting in data loss. (**b**) With application of filtering schemes, data from more patients have been accommodated (up to 20 patients). (**c**) The total data generated (from 5 patients in 5 min) reduce significantly as more and more filtering schemes are applied. (**d**) Shows the gains in percent (reduction in data volume per patient per second) when filtering schemes are applied in standalone mode and in combination. The experiment was performed on 5 patients.

When not applying any filtering, each patient generates 2 megabits of data per second and creates pressure on the network. We examined the collected data to reduce the pressure of data (from IoT devices) on the network and support a larger number of patients (or sensors). A close inspection of data in Table 3 reveals that some sensors show redundant values (temperature, light, pressure etc.). This creates room for the application of fog computing at this IoT device level. With the dropping of redundant values (as shown in experiment 3 in Table 4), we were able to support data from 7 patients on a 10 Mbps connection. A sample of data from the experiment, as shown in Table 3, shows that redundant data for all sensors are dropped (at the IoT device level). This results in reducing the data from each patient to about 1.6 megabits per second, reducing the data pressure by 20% as shown in Figure 5. Further, the details of each filtering scheme are shown in Table 5.

The dropping of data gives us gains in reducing the amount of data generated by the IoT device. However, there is room for more filtering at the device level. A further examination of data in Table 2 reveals that some of the data have less variation (temperature, pressure, etc.) in some of their readings compared to other indicators (accelerometer, gyroscope, etc.). This means that we can define a threshold difference (in comparison to the previous value) and safely drop the entries that do not pass the threshold difference (we set a threshold difference of 0.10 for the temperature, light and pressure). Applying this filtering in conjunction with dropping duplicate values, we performed an experiment (experiment 4). The data (generated per patient per second) were significantly lower (1.4 megabits per second) compared to when not using any filtering (2 megabits per second). This resulted in a gain of 30%, and we were able to support 7 patients on a 10 Mbps network bandwidth as shown in Figure 5.

As part of the effort to further reduce the volume of generated data and potentially support a larger number of patients, we further analyze the data. A further examination of our sensors indicates that some of them have less variation in their values compared to other sensors. This means that those indicators’ values change less frequently than others. For example, values of temperature, light, pressure, magnetometer, and humidity have less variation in their values compared to other indicators, such as accelerometer, gyroscope and sound. Also, some indicators (like pressure, humidity and light) may be of lesser importance than other indicators (like the patient’s movement). This gives a useful insight that the same sampling frequency may not be required for all indicators, opening room for the application of a variable sampling frequency on different sensors. Taking this opportunity, we use Equations (1) and (2) ($patient_{condition}=1$, $sensor_{sensitivity}=0.5$) and apply a sampling frequency of 500 samples per second to the less variant sensors, while leaving the others unchanged as shown in Table 6. The application of variable sampling gave us a combined gain of 60% and reduced the amount of data (generated per patient) to 0.8 Megabits per second. This way, we are able to support a maximum of 12 patients on a 10 Mbps network connection.

For an experiment that lasted five minutes (on ten patients), the total data volume was reduced from 6.3 GB (when not applying any edge processing) to 2.5 GB (with filtering and usage of different sampling frequencies). The gain significantly reduces the pressure on the network, cloud storage and computing services.

### 5.3. Effect of Edge Computing at Smartphone Level

The data collection app receives the data from the IoT device using the BLE module. The app combines the timestamp and GPS (latitude and longitude) with it and sends it to the cloud with the unique device ID (which uniquely identifies the patient) using the API call.

We face some issues with data collection and its transmission to local app elements on high frequencies. We observe some undefined numbers for some sensors and also observe that sometimes packets are dropped.

At the app level, we apply a second level of fog computing. This will further reduce the data pressure on the network and help raise early alarms about vital patient conditions. At this level, the data for different indicators are checked to determine if they fall in the normal range. For example, for temperature, the normal range is defined as 30–34 °C. We also define similar normal ranges for other indicators (light, pressure, vibration, etc.). Anything that falls in these ranges is dropped and not forwarded to the cloud. Using this approach, we see a further 29% reduction in data volume as shown in Table 7. This volume might differ significantly depending on the normal ranges and the condition of the patients. The data volume per patient (per second) is reduced to 0.24 megabits (given that we are applying all the filtering schemes. The combined gains from the three filtering schemes are now able to support 20 patients on a 10 Mbps network bandwidth as shown in Figure 5. Fog computing could be applied to generate notifications or alerts at this early stage of data handling.

It is essential to mention that the gains in reducing data flow are translated into reducing the pressure on data stores (relational and non-relational) and other cloud-processing applications.

Further, multiplexing is applied to prepare larger packets. The multiplexing does not help reduce the amount of data; however, it helps reduce the number of calls to the cloud API gateway. Reducing the number of calls is helpful when the costs are associated with cloud server spins (in asynchronous demand-driven modes). Remember that multiplexing can be adaptive in proportion to the server costs and the importance of patient indicators.

### 5.4. Utility of API Gateways

The cloud gateway for incoming data successfully reads the data and generated alerts. Every API call has attached a function on the lambda server on the AWS cloud. The lambda function tokenizes the values received through the API gateway. Once the data are available to the function, the device id is used as a key to identify the patient and fetch his/her information from the relational data store. The lambda processing function performs two types of operations on the received data. In the first type of processing, the data are analyzed against certain thresholds for every health parameter, and if a value is marked, the lambda function triggers the corresponding guardian and physician’s alerts using the AWS SNS application. The SNS application is connected to Firebase Cloud Messaging. This shows the lambda functions’ ability to pre-process the received data and generate alerts.

The second type of processing involves invoking different services on the cloud. For example, an audio sample of a patient’s heartbeat may be used to trigger an artificial intelligence or machine learning application. The data for these services are first stored in S3, and after post-processing, the updates are saved to RDS.

### 5.5. Utility of Physician and Caretaker Apps

The guardians and physicians receive the alerts on their smartphones, on which application dashboards are installed. These alerts are shown in the notification panel with their priority set to high. The guardians and physicians request to see the patient’s health history and visualize it to make it more readable and understandable as shown in Figure 4c.

### 5.6. Reliability of Overall Model

The reliability of the overall model was studied from the perspective of data collection, correctness, data transmission, system alerts, and usability. We collected the data using TI Sensor Tag cc3250 and found it very accurate, as we compared with data collected using other IoT devices. It was computed using Equation (3) and was found to be more than 95% for all the sensors:

(3) $$Sensor_{accuracy}=\frac{\sum_{j=1}^{n}\sum_{k=1}^{m}\left|SensorTag_{j,k}-RefDevice_{j,k}\right|*100}{N}$$

where $SensorTag_{j,k}$ refers to sample *k* of sensor *j* on the Sensor Tag, while $RefDevice_{j,k}$ refers to sample *k* of sensor *j* on the reference device.

We also encountered some sample dropping on very high frequencies (1000 samples per second) and saw undefined values for some of the sensors. In total, three million measurements were taken with an effectiveness of 85%. This means that for 15% of the samples, the sensors were unable to show valid numbers. The undefined readings mostly came from light and pressure sensors. In practice, we seldom use the mentioned sensor at such high frequencies.

An issue that we encountered with the IoT module was the battery power. The battery lasted for about 10 h when used at a frequency of 1000 samples per second with all ten sensors enabled. Overall, the model was quite effective in providing accurate remote health data. The different model reliability measures identify important error sources in remote monitoring models. Our model perform better in comparison to the system presented by [45].

The data were transmitted to the cloud using http requested, and 96% of the data were received in the cloud. We observed some packet loses, especially with data from all patients (with all sensors generating data at 1000 samples per second). The network bandwidth became congested, and we observed some packet losses en route to the cloud.

We generated some notifications at the cloud level from an asynchronous server, and all alerts were received by the receivers at their apps. We tested the model for the sensing of environmental parameters; however, it is equally applicable to the monitoring of patients with different needs.

## 6. Discussion and Conclusions

This paper presents a comprehensive, cloud-supported model for patient monitoring that seamlessly integrates IoT, computational engines, and application programs through well-defined interfaces. The model addresses various aspects of remote patient monitoring, including data collection, the efficient handling of large volumes of data, transmission to the cloud, data storage and processing, and effective communication with applications.

Our model’s standout feature is its end-to-end solution, providing a comprehensive and integrated approach to remote patient monitoring. Unlike other proposed solutions that focus on specific aspects, our model holistically covers the entire monitoring process. Its modular design delineates into three well-defined modules: sensing, network, and application.

The sensing module, which is foundational to data collection from IoT devices, incorporates advanced edge processing techniques for the efficient handling of the large data volumes generated by sensors. By using filtering, variable sampling, and multiplexing techniques, our model significantly reduces the volume of sensor-generated data, showcasing improved scalability that accommodates four times more patients compared to traditional approaches without these edge processing capabilities. For instance, in a testing trial, our model successfully monitored 40 patients simultaneously, providing real-time data insights into their vital signs and environmental parameters.

The network module, housed in the cloud, integrates seamlessly with the sensing module through well-defined APIs. It offers extensive possibilities for data processing, sharing, and utilization, with components including API gateways, data stores, and processing modules. We envision an enhancement in the model’s effectiveness through the integration of AI and ML services, enabling advanced data analysis, decision making, and personalized healthcare interventions. For instance, leveraging ML algorithms could detect anomalies in patients’ vital signs, alerting healthcare providers to potential health risks in real time.

The application module establishes a seamless connection between physicians, patients, and their caretakers through user-friendly interfaces, such as web dashboards and smartphone applications. The modular architecture of our model allows for the independent scaling of components within each module, ensuring flexibility and adaptability to evolving needs.

In conclusion, our work offers a robust and comprehensive model for remote patient monitoring, efficiently handling vast amounts of data from IoT devices. The model’s end-to-end solution, along with its modularity and advanced edge processing techniques, contributes to improved efficiency, scalability, and patient management.

Moving forward, our commitment to continuous improvement has illuminated potential future avenues of research. This includes exploring the integration of ML and AI modules, which holds significant promise for enhancing data analysis, decision making, and personalized interventions in our model. We are also focusing on facilitating real-world testing in collaboration with healthcare consultants and providers to further refine our model’s practical application. At the same time, we place high importance on data privacy and are taking strides to ensure our platform’s full compliance with GDPR and other relevant data protection standards.

Additional future directions include further analyzing and integrating AI and ML modules to elaborate the model’s effectiveness, exploring the clinical aspects of collected data, and expanding its capabilities in monitoring additional health indicators. Also, increasing the number of patients that our model can handle and investigating the impact of multi-node gateways and load balancing are promising directions for our future research.

## Figures and Tables

**Table 2:** A sample from data recorded from 5 patients using Sensor Tag cc2650. The data show redundancy in values for different sensors (temperature, pressure, humidity, etc.)

Sample	Temperature (°C)	Ax (m/s${}^{2}$)	Gx (deg/s)	Pressure (Pa)	Humidity (%)	Sound (db)	Light (lux)	Time
1	36.42	−0.605712891	−0.129699707	954.3	20.9	61.2	26.57	18:25:10.23
2	36.42	−0.013427734	0.183105469	954.29	21.3	62.3	26.57	18:25:10.25
3	36.42	−0.200683594	1.914978027	954.3	21.4	48.2	26.25	18:25:10.26
4	36.42	0.035400391	−2.517700195	954.33	21.4	39.7	26.49	18:25:10.26
5	36.42	0.251953125	−1.864807129	954.33	21.4	63.8	23.59	18:25:10.27
6	36.47	0.086914063	−1.480102539	954.33	21.4	61.2	26.49	18:25:10.28
7	36.49	0.143798828	0.788513184	954.35	21.5	58.7	23.52	18:25:10.28
8	36.49	−1.482421875	−1.202697754	954.35	21.5	66.3	23.75	18:25:10.28
9	36.49	0.175537109	−2.554199219	954.31	21.5	62.5	23.67	18:25:10.30
10	36.36	−0.616943359	−6.629943848	954.32	21.5	44.3	23.67	18:25:10.31
11	36.36	0.552978516	−1.037597656	954.31	21.4	36.6	23.6	18:25:10.32
12	36.36	1.440429688	2.004333496	954.31	21.4	36.4	23.6	18:25:10.33
13	36.36	1.386474609	3.517150879	954.31	21.4	38.5	23.67	18:25:10.34
14	36.41	−0.953125	−0.470825195	954.29	21.4	37.4	23.6	18:25:10.37

**Table 3:** Applying filtering on IoT data in Table 2 and removing redundant data of temperature, pressure, humidity, and light gives a 47% reduction in the number of readings. The vibration, tilt and sound vary more frequently and do not have any redundant values.

Sample	Temperature (°C)	Ax (m/s${}^{2}$)	Gx (deg/s)	Pressure (Pa)	Humidity (%)	Sound (db)	Light (lux)	Time
1	36.42	−0.605712891	−0.129699707	954.3	20.9	61.2	26.57	18:25:10.23
2		−0.013427734	0.183105469	954.29	21.3	62.3		18:25:10.25
3		−0.200683594	1.914978027	954.3	21.4	48.2	26.25	18:25:10.26
4		0.035400391	−2.517700195	954.33		39.7	26.49	18:25:10.26
5		0.251953125	−1.864807129			63.8	23.59	18:25:10.27
6	36.47	0.086914063	−1.480102539			61.2	26.49	18:25:10.28
7	36.49	0.143798828	0.788513184	954.35		58.7	23.52	18:25:10.28
8		−1.482421875	−1.202697754		21.5	66.3	23.75	18:25:10.28
9		0.175537109	−2.554199219	954.31		62.5	23.67	18:25:10.30
10	36.36	−0.616943359	−6.629943848	954.32		44.3		18:25:10.31
11		0.552978516	−1.037597656	954.31	21.4	36.6	23.6	18:25:10.32
12		1.440429688	2.004333496			36.4		18:25:10.33
13		1.386474609	3.517150879			38.5	23.67	18:25:10.34
14	36.41	−0.953125	−0.470825195	954.29		37.4	23.6	18:25:10.37

**Table 4:** All the experiments were performed on a 10 Mbps network bandwidth with all 10 sensors of the IoT device enabled.

Experiment No.	Data Generated per Second (Megabits)	No. of Patients /SensorTags	Sampling Rate (per Second)	Filtering 1	Filtering 2	Filtering 3	Filtering 4	Remarks
1	21	10	1000	No	No	No	No	Large network contention, packets dropping
2	10.4	5	1000	No	No	No	No	Little network contention
3	9.5	7	1000	Yes	No	No	No	No network issues
4	9.6	8	1000, 500	Yes	Yes	No	No	No network issues
5	9.2	10	1000, 500	Yes	Yes	Yes	No	No network issues
6	9.3	20	1000, 500	Yes	Yes	Yes	Yes	No network issues

**Table 5:** A list of different filtering techniques that were applied at the IoT and mobile edge levels.

Filtering Label	Description	Applied At
Filtering 1	remove duplicates only	IoT device level
Filtering 2	remove duplicates and data in threshold	IoT device level
Filtering 3	variable sampling	IoT device level
Filtering 4	mobile edge computing	Mobile edge level

**Table 6:** A sample from data recorded using Sensor Tag cc2650. The experiment employs a first level of filtering (which is the dropping of an attribute value if it is the same as its previous reading).

Sample	Temperature (°C)	Ax (m/s${}^{2}$)	Gx (deg/s)	Pressure (Pa)	Humidity (%)	Sound (db)	Light (lux)	Time
1	35.51	−0.614746094	−0.89642334	954.33	21.1	63.1	26.65	14:33:19.45
2	32.25	−0.061035156	−1.32910156		21.3	63.3	26.66	14:33:19.45
3		−0.019287109	1.2053833		21.5	60.6		14:33:19.46
4		0.578369141	−1.96887207	954.32		56.2		14:33:19.48
5		0.000488281	−0.991821289	954.31		54.2		14:33:19.49
6	31.17	0.467041016	0.42553711	954.29		41.5		14:33:19.49
7		0.331542969	1.64300537	954.31	21.6	42.5	26.81	14:33:19.50
8		−0.06640625	2.262084961		21.4	68.7	26.73	14:33:19.50
9	33.51	−0.583251953	2.950378418			69.4	26.66	14:33:19.51
10	32.49	−0.528564453	−1.49639893	954.29	21.7	64.3	26.45	14:33:19.51
11	34.16	−0.40234375	0.91217041	954.34		61.7	26.66	14:33:19.54
12		1.070068359	−1.185791016		21.3	52.6		14:33:19.55
13		0.396484375	0.2130127		21.4	48.3	26.73	14:33:19.55
14	35.45	−0.381103516	1.799438477			47.4		14:33:19.56

**Table 7:** Applying filtering on IoT data in Table 3 and dropping data that fall in the normal ranges for temperature, pressure, humidity, and light sensors gives a 56% reduction in the number of readings. The variation in vibration, tilt and sound is large, and we do not apply this level of filtering on them.

Sample	Temperature (°C)	Ax (m/$s^{2}$)	Gx (deg/s)	Pressure (Pa)	Humidity (%)	Sound (db)	Light (lux)	Time
1	35.51	−0.614746094	−0.89642334	954.33	21.1	63.1	26.65	14:33:19.45
2		−0.061035156	−1.32910156			63.3		14:33:19.45
3		−0.019287109	1.2053833		21.5	60.6		14:33:19.46
4		0.578369141	−1.96887207			56.2		14:33:19.48
5		0.000488281	−0.991821289	954.31		54.2		14:33:19.49
6		0.467041016	0.42553711			41.5		14:33:19.49
7		0.331542969	1.64300537	954.31	21.6	42.5	26.81	14:33:19.50
8		−0.06640625	2.262084961			68.7		14:33:19.50
9	34.27	−0.583251953	2.950378418			69.4	26.66	14:33:19.51
10		−0.528564453	−1.49639893			64.3		14:33:19.51
11	34.16	−0.40234375	0.91217041	954.34		61.7	26.66	14:33:19.54
12		1.070068359	−1.185791016			52.6		14:33:19.55
13		0.396484375	0.2130127		21.4	48.3	26.73	14:33:19.55
14	35.45	−0.381103516	1.799438477			47.4		14:33:19.56

## Data Availability

Not applicable.
